# Building Dual AI Models and Nomograms Using Noninvasive Parameters for Aiding Male Bladder Outlet Obstruction Diagnosis and Minimizing the Need for Invasive Video-Urodynamic Studies: Development and Validation Study

**DOI:** 10.2196/58599

**Published:** 2024-07-23

**Authors:** Chung-You Tsai, Jing-Hui Tian, Chien-Cheng Lee, Hann-Chorng Kuo

**Affiliations:** 1 Divisions of Urology, Department of Surgery, Far Eastern Memorial Hospital New Taipei Taiwan; 2 Department of Electrical Engineering, Yuan Ze University Taoyuan Taiwan; 3 Department of Medical Research, Hualien Tzu Chi Hospital, Buddhist Tzu Chi Medical Foundation Hualien Taiwan; 4 Department of Urology, Hualien Tzu Chi Hospital, Buddhist Tzu Chi Medical Foundation and Tzu Chi University Hualien Taiwan

**Keywords:** bladder outlet obstruction, lower urinary tract symptoms, machine learning, nomogram, artificial intelligence, video urodynamic study

## Abstract

**Background:**

Diagnosing underlying causes of nonneurogenic male lower urinary tract symptoms associated with bladder outlet obstruction (BOO) is challenging. Video-urodynamic studies (VUDS) and pressure-flow studies (PFS) are both invasive diagnostic methods for BOO. VUDS can more precisely differentiate etiologies of male BOO, such as benign prostatic obstruction, primary bladder neck obstruction, and dysfunctional voiding, potentially outperforming PFS.

**Objective:**

These examinations’ invasive nature highlights the need for developing noninvasive predictive models to facilitate BOO diagnosis and reduce the necessity for invasive procedures.

**Methods:**

We conducted a retrospective study with a cohort of men with medication-refractory, nonneurogenic lower urinary tract symptoms suspected of BOO who underwent VUDS from 2001 to 2022. In total, 2 BOO predictive models were developed—1 based on the International Continence Society’s definition (International Continence Society–defined bladder outlet obstruction; ICS-BOO) and the other on video-urodynamic studies–diagnosed bladder outlet obstruction (VBOO). The patient cohort was randomly split into training and test sets for analysis. A total of 6 machine learning algorithms, including logistic regression, were used for model development. During model development, we first performed development validation using repeated 5-fold cross-validation on the training set and then test validation to assess the model’s performance on an independent test set. Both models were implemented as paper-based nomograms and integrated into a web-based artificial intelligence prediction tool to aid clinical decision-making.

**Results:**

Among 307 patients, 26.7% (n=82) met the ICS-BOO criteria, while 82.1% (n=252) were diagnosed with VBOO. The ICS-BOO prediction model had a mean area under the receiver operating characteristic curve (AUC) of 0.74 (SD 0.09) and mean accuracy of 0.76 (SD 0.04) in development validation and AUC and accuracy of 0.86 and 0.77, respectively, in test validation. The VBOO prediction model yielded a mean AUC of 0.71 (SD 0.06) and mean accuracy of 0.77 (SD 0.06) internally, with AUC and accuracy of 0.72 and 0.76, respectively, externally. When both models’ predictions are applied to the same patient, their combined insights can significantly enhance clinical decision-making and simplify the diagnostic pathway. By the dual-model prediction approach, if both models positively predict BOO, suggesting all cases actually resulted from medication-refractory primary bladder neck obstruction or benign prostatic obstruction, surgical intervention may be considered. Thus, VUDS might be unnecessary for 100 (32.6%) patients. Conversely, when ICS-BOO predictions are negative but VBOO predictions are positive, indicating varied etiology, VUDS rather than PFS is advised for precise diagnosis and guiding subsequent therapy, accurately identifying 51.1% (47/92) of patients for VUDS.

**Conclusions:**

The 2 machine learning models predicting ICS-BOO and VBOO, based on 6 noninvasive clinical parameters, demonstrate commendable discrimination performance. Using the dual-model prediction approach, when both models predict positively, VUDS may be avoided, assisting in male BOO diagnosis and reducing the need for such invasive procedures.

## Introduction

Diagnosing the underlying causes of nonneurogenic male lower urinary tract symptoms (LUTS) associated with bladder outlet obstruction (BOO) is complex and cannot rely solely on symptomatology [1]. The pathophysiology of male BOO encompasses both anatomical and functional obstruction subtypes, such as benign prostatic obstruction (BPO), urethral stricture (US), primary bladder neck obstruction (PBNO), and dysfunctional voiding (DV) [2]. To accurately determine the pathophysiology behind BOO, it is crucial to conduct a comprehensive urodynamic study (UDS). These studies include 2 invasive diagnostic approaches—pressure-flow studies (PFS) or video-urodynamic studies (VUDS). PFS assesses bladder and outlet dynamics during urination [3,4]. Nevertheless, VUDS is a more precise and advanced examination that synchronously records PFS data plus real-time fluoroscopic imaging (x-ray) during both the storage and emptying phases of the bladder, providing a more detailed understanding of the underlying dysfunction with both functional and anatomical insights [5,6]. However, due to the higher costs associated with VUDS, as well as the requirements for specialized x-ray equipment and radiation protection, it is typically performed in tertiary medical centers and is not routinely available in all hospital settings.

PFS is crucial for evaluating bladder outlet dynamics with measurements of bladder pressure and urinary flow. According to the International Continence Society (ICS), male BOO is confirmed when the BOO index reaches 40 or higher (detailed in the “Methods” section) [7]. However, PFS falls short in distinguishing specific pathophysiologies such as PBNO or DV. VUDS, in contrast, offers a more comprehensive analysis of male LUTS suggestive of BOO, particularly in cases of PBNO or DV [8]. PBNO involves the failure of the bladder neck’s smooth muscle to adequately open during voiding. In contrast, DV is characterized by an intermittent or fluctuating urinary flow, typically due to inadequate or inconsistent relaxation of the external urethral sphincter during the process of voiding in men who do not have neurological disorders [7]. This distinction between PBNO and DV is critical since their treatment strategies differ markedly [9,10]. Beyond medication, PBNO often necessitates surgical intervention, whereas DV is primarily managed with behavioral therapies and physical therapies instead of surgery, highlighting the need for precise diagnostic methods.

From the clinical decision-making perspective, there exists a dilemma for men with medication-refractory nonneurogenic LUTS who are suspected of having BOO. The following question arises: which diagnostic UDS is most appropriate for the patient? (1) Should one take the first pathway and perform PFS only to potentially obtain an accurate diagnosis? (2) Or should one follow the second pathway, starting with PFS and proceeding to VUDS if a definitive diagnosis is not achieved? (3) Alternatively, is the third pathway preferable, where one directly conducts VUDS for a detailed diagnosis?

Given the invasive nature of PFS and VUDS, which can lead to discomfort and the risk of urinary tract infection, their use may not be feasible for all men with LUTS. These procedures are especially impractical for individuals who are physically frail. With these considerations, it is desirable to develop artificial intelligence (AI) models that leverage noninvasive clinical parameters to aid in the decision-making process. The aim is to predict the possible pathophysiology of the patient, reducing the need for invasive examinations, or to precisely determine which patients require VUDS. This approach would reserve VUDS for those who would benefit most from its detailed analysis. Although noninvasive methods and parameters have been proposed to predict the likelihood of BOO in male common or treatment-naïve LUTS [11-17], to our knowledge, no existing studies have developed prediction models using VUDS as the diagnostic standard, especially for those with medication-refractory nonneurogenic LUTS suspected of BOO. Addressing this gap, our goal is to develop and validate 2 clinical nomograms and AI models that can predict both International Continence Society–defined bladder outlet obstruction (ICS-BOO) and video-urodynamic studies–diagnosed bladder outlet obstruction (VBOO) using only noninvasive clinical parameters, potentially streamlining the diagnostic process for this patient group.

## Methods

### Overview

**Figure 1:**
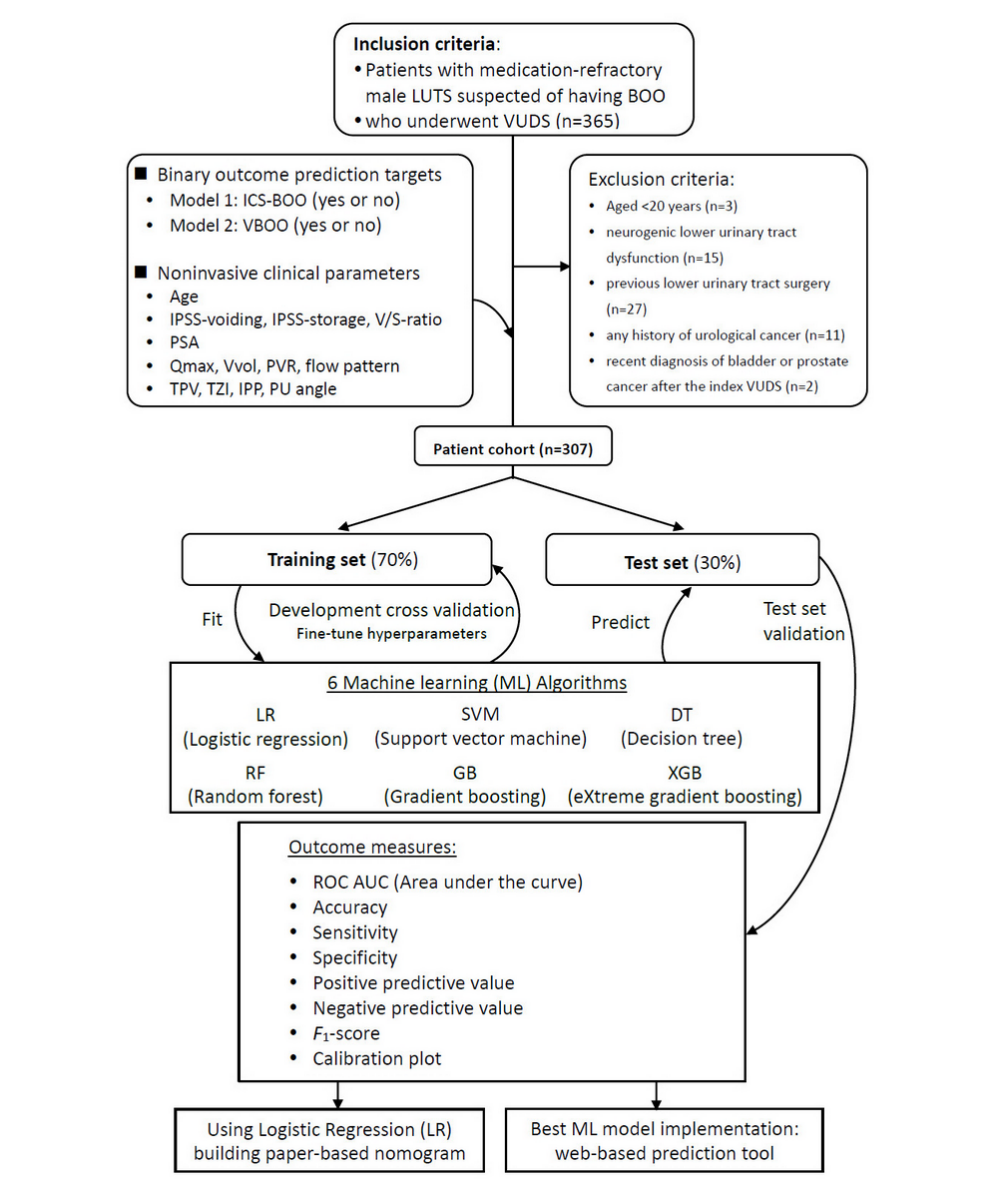
Study flow diagram and methodological framework for bladder outlet obstruction predictive model development. This diagram illustrates the methodological approach to constructing predictive models for bladder outlet obstruction. BOO: bladder outlet obstruction; ICS-BOO: International Continence Society–defined bladder outlet obstruction; IPP: intravesical prostatic protrusion; IPSS: International Prostate Symptom Score; LUTS: lower urinary tract symptoms; PSA: prostate-specific antigen; PU angle: prostatic urethral angle; PVR: postvoid residual; Qmax: maximum urinary flow rate; ROC: receiver operating characteristic; TPV: total prostate volume; TZI: transitional zone index; V/S ratio: voiding to storage score ratio; VBOO: video-urodynamic studies–diagnosed bladder outlet obstruction; VUDS: video-urodynamic studies; Vvol: voided volume.

The study flow diagram and methodological framework for the development of BOO predictive models are illustrated in Figure 1. This outlines the process from the initial patient selection based on inclusion and exclusion criteria, through the development and test validation of various machine learning (ML) algorithms, to the final deployment in the form of both paper-based nomograms and an integrated web-based clinical decision support tool.

### Patient Population

This retrospective study encompassed a cohort of male patients with medication-refractory, nonneurogenic LUTS who were suspected of having BOO and underwent VUDS at a single medical center from January 2001 to May 2022.

The inclusion criteria targeted men who had been on continuous medication for over 3 months without a response to at least one type of LUTS treatment, including alpha-blockers, 5-alpha-reductase inhibitors, or antimuscarinics, before undergoing VUDS (Multimedia Appendix 1). Exclusion criteria included patients younger than 20 years, those with neurogenic lower urinary tract dysfunction, previous lower urinary tract surgery, any history of urological cancer, or a recent diagnosis of bladder or prostate cancer after the index VUDS.

### Noninvasive Variables and VUDS Procedure

In daily clinical practice for men with LUTS, we routinely conduct history-taking, physical examinations, and record the International Prostate Symptom Score (IPSS). We also measure prostate-specific antigen levels and perform a transrectal ultrasound of the prostate along with uroflowmetry. The noninvasive clinical parameters chosen to construct the predictive models, serving as features for ML, are derived from these evaluations. These parameters include uroflowmetry measures such as maximum urinary flow rate (Qmax; measured in mL/s), voided volume (Vvol; measured in mL), postvoid residual (measured in mL), and flow pattern (qualitatively assessed). IPSS components comprise IPSS-voiding, IPSS-storage, and the IPSS-voiding to storage score ratio. Transrectal ultrasound of the prostate measures include total prostate volume (TPV; measured in mL), transitional zone index (defined as the percentage of transitional zone volume to TPV), intravesical prostatic protrusion (IPP; measured in cm), and prostatic urethral angle (PUA; measured in degrees).

During the VUDS procedure, we recorded bladder sensation, capacity, compliance, detrusor activity, and the maximum detrusor pressure at Qmax (Pdet.Qmax). In addition, electromyography and fluoroscopy imaging (x-ray) during both the storage and voiding phases were recorded using the Laborie system. All VUDS reports were collected and reviewed by a single urology professor (HCK) with more than 30 years of experience in VUDS to ensure consistency and reliability in the interpretation of these complex studies.

### Definition of 2 Prediction Targets

In this study, 2 distinct prediction targets were defined for the development of the predictive models. The first model, “model 1,” is designed to predict ICS-BOO as a binary outcome. This prediction is based on the ICS’s criteria for the bladder outlet obstruction index (BOOI) [7], also referred to as the Abrams-Griffiths number. The BOOI is calculated using the formula “Pdet.Qmax-2×Qmax.” A BOOI threshold value of 40 or higher is defined as obstructive, thus identifying the presence of ICS-BOO. Furthermore, a BOOI between 20 and 40 falls into an equivocal zone according to the ICS’s criteria.

The second model, “model 2,” is developed to predict VBOO as a binary outcome. VBOO includes a range of conditions contributing to male BOO, such as BPO, PBNO, DV, and US. Both PBNO and DV are types of functional BOO, as detailed in sections Bladder Outlet Obstruction (BOO), Dysfunctional Voiding, Detrusor Sphincter Dyssynergia, and Primary Bladder Neck Obstruction (nonneurogenic) of the ICS Standardisation Report for adult male LUTS [7]. The ICS Standardisation Reports also indicate that VUDS is necessary for diagnosing PBNO or DV, as these conditions cannot be adequately identified by PFS alone [7]. Some types of PBNO or DV would not reveal a classical “high-pressure, low-flow pattern.” This means that patients with a BOOI<40, though not meeting the ICS-BOO criteria, could still be diagnosed with PBNO or DV as BOO through VUDS. Consequently, it is possible that a larger number of patients within the same cohort may be diagnosed with VBOO compared to ICS-BOO.

### Model Derivations With Development and Test Validation

In the derivation of our predictive models, the data set from the entire cohort was randomly split once, allocating 70% to the training set and 30% to the test set to support both development and test validation processes. To develop the 2 prediction models, ML approaches using logistic regression (LR) were used. Variable selection for these models was conducted using a backward stepwise approach based on Akaike’s information criterion (AIC). The β coefficients from the final regression models were then used to construct nomograms, which provide a paper-based, visual representation of the prediction models and facilitate their application in clinical practice. In addition, web-based prediction tools were also deployed to assist in AI-driven decision-making.

The development validation was rigorously conducted using a repeated 5-fold cross-validation technique within the training set. To ensure the robustness of development validation, the process was repeated 20 times, resulting in 100 iterations of model training and validation. The performance of the models was then evaluated based on the average metrics from these iterations, providing an unbiased estimation of model performance.

For test validation, the best model and hyperparameters derived from the development validation were retrained on the entire training data set and then tested on the independent test set to assess their generalizability and performance in a test cohort. This comprehensive validation approach aimed to bolster the robustness of the predictive models before their potential clinical application.

In addition to LR, 5 other ML algorithms were used to construct models using the same methodology as described previously. These algorithms included support vector machine, decision tree, random forest, gradient boosting, and extreme gradient boosting. Each algorithm was systematically evaluated to assess its performance in predicting BOO, ensuring a comprehensive analysis of different modeling approaches.

### Outcome Measures and Statistical Analysis

In assessing the performance of our predictive models, we used several outcome measures to evaluate their effectiveness. Discrimination (the model’s ability to distinguish between the presence and absence of an outcome) was measured by the area under the receiver operating characteristic curve (AUC). The AUC provides a single measure summarizing the model’s accuracy across all classification thresholds. The confusion matrix provided crucial metrics influenced by the chosen cut-off value, such as accuracy, sensitivity, specificity, positive predictive value (PPV), negative predictive value (NPV), and the *F*_1_-score. The optimal threshold was determined using the Youden index. Model agreement was assessed using calibration plots, which visually compare predicted probabilities with observed outcomes, thus ensuring that the model’s predictions align closely with actual risk. The Hosmer-Lemeshow test was also used to evaluate the goodness-of-fit of the models [18]. A *P* value of less than .05 was considered statistically significant.

Numerical variables with a normal distribution are reported as means and SDs and compared using an independent *t* test (2-tailed). All categorical demographic variables were compared using the chi-square test. LR models and nomograms were developed using R (version 4.1.1; R Core Team) with the “glmnet” and “rms” packages for model fitting and nomogram visualization [19]. Python’s “scikit-learn” library (version 0.24.2) was used for supplementary ML and validation tasks [20]. In addition, SPSS Statistics (version 25; IBM Corp) was used for statistical testing [21].

### Ethical Considerations

The study protocol was approved by the Research Ethics Committee of Hualien Tzu Chi Hospital, Buddhist Tzu Chi Medical Foundation, on June 15, 2022 (IRB111-132-B).

## Results

### Patient Cohort Characteristics and LUTS Diagnoses

This study analyzed a cohort of 307 male patients presenting with medication-refractory nonneurogenic LUTS suspected of BOO, all of whom underwent VUDS. The patient group was further subdivided into a training set of 70% (215) and a test set of 30% (92). The average age of the entire cohort was 67.8 (SD 9.7, range 32.2-91.5) years, which did not differ significantly between the training and test groups (*P*=.93). Clinical parameters such as IPSS-voiding, IPSS-storage scores, IPSS-voiding to storage score ratio, Qmax, Vvol, postvoid residual, TPV, transitional zone index, IPP, and PUA all showed no statistically significant difference when the training set was compared with the test set (Table 1).

**Table 1:** Characteristics, distribution of video-urodynamic studies diagnoses, and bladder outlet obstruction binary classifications in the patient cohort, with training or test set split.

Cohort								Entire					Training					Test			Train vs test *P* value		
Overall, n (%)								307 (100)					215 (70)					92 (30)			—^a^		
**Characteristics, mean (SD)**																							
	Age (years)						67.8 (9.7), range (32.2-91.5)					67.8 (9.8), range (38.2-91.5)					67.9 (9.5), range (32.2-86.9)			.93^b^			
	IPSS^c^-voiding						9.5 (5.9)					9.5 (5.7)					9.7 (6.3)			.78^b^			
	IPSS-storage						8.7 (4.1)					8.6 (4)					9 (4.2)			.45^b^			
	IPSS-V/S^d^ ratio						1.5 (1.6)					1.5 (1.6)					1.5 (1.7)			.99^b^			
	Qmax^e^ (mL/s)						10.2 (6.5)					10.6 (7)					9.4 (5.1)			.11^b^			
	Voided volume (mL)						196.8 (124.1)					200.1 (124.7)					189.2 (123)			.48^b^			
	Postvoid residual (mL)						44.1 (83.9)					48.7 (96.3)					33.5 (41.2)			.15^b^			
	Total prostate volume (mL)						38.1 (19.5)					39.2 (20.9)					35.4 (15.7)			.12^b^			
	Transitional zone index						0.4 (0.16)					0.4 (0.16)					0.42 (0.14)			.39^b^			
	IPP^f^ (cm)						0.3 (0.6)					0.3 (0.6)					0.3 (0.5)			.39^b^			
	Prostatic urethral angle (º)						26.3 (18.7)					25.9 (18.8)					27.4 (18.3)			.52^b^			
**VUDS^g^ diagnosis, n (interclass%)**																							0.50^h^
	**Obstruction-predominant**																						
				Primary bladder neck obstruction		120 (39.1)					84 (39.1)					36 (39.1)			—				
				Benign prostatic obstruction		87 (28.3)					61 (28.4)					26 (28.3)			—				
				Dysfunctional voiding		45 (14.7)					32 (14.9)					13 (14.1)			—				
				Urethral stricture		0 (0)					0 (0)					0 (0)			—				
	**Bladder dysfunction**																						
					Detrusor underactivity		13 (4.2)			1 (1.1)					12 (5.6)						—		
					Detrusor overactivity		28 (9.1)			10 (10.9)					18 (8.4)						—		
					Bladder oversensitivity		6 (2)			2 (2.2)					4 (1.9)						—		
					Generally normal		8 (2.6)			4 (1.9)					4 (4.3)						—		
**BOO^i^ binary classifications, n (interclass%)**																							
	**ICS-BOO^j^**																					0.872^h^	
			Yes				82 (26.7)			58 (27)					24 (26.1)						—		
			No				225 (73.3)			157 (73)					68 (73.9)						—		
	**VBOO^k^**																					0.866^h^	
		Yes				252 (82.1)			177 (82.3)					75 (81.5)						—			
		No				55 (17.9)			38 (17.7)					17 (18.5)						—			

^a^Not applicable.

^b^Comparison between training or test groups using independent *t* test.

^c^IPSS: International Prostate Symptom Score.

^d^IPSS-V/S ratio: International Prostate Symptom Score voiding to storage score ratio.

^e^Qmax: maximum urinary flow rate.

^f^IPP: intravesical prostatic protrusion.

^g^VUDS: video-urodynamic studies.

^h^Comparison between training or test groups using chi-square test.

^i^BOO: bladder outlet obstruction.

^j^ICS-BOO: International Continence Society–defined bladder outlet obstruction.

^k^VBOO: video-urodynamic studies–diagnosed bladder outlet obstruction.

In the breakdown of VUDS diagnoses within the cohort, obstruction-predominant conditions were observed, with PBNO accounting for 39.1% (120/307), BPO for 28.3% (87/307), and DV for 14.7% (45/307) of the cases. Notably, there were no instances of US reported. Regarding bladder dysfunction, detrusor underactivity (DU) was present in 4.2% (13/307) of the patients, detrusor overactivity (DO) in 9.1% (28/307), and bladder oversensitivity in 2% (6/307), with 2.6% (8/307) of the cohort deemed generally normal. The distribution of these diagnoses was consistent across both training and test sets, as indicated by the nonsignificant chi-square *P* values, demonstrating a homogeneous division of conditions between the 2 groups (Table 1).

For the ICS-BOO, only 26.7% (82/307) of the entire cohort was classified as having BOO. However, the prevalence of VBOO was much higher, with 82.1% (225/307) of patients being identified as having BOO (McNemar test *P*<.001). This disparity in BOO prevalence between ICS-BOO and VBOO highlights the different diagnostic criteria and approaches between PFS and VUDS. VUDS excels in identifying the causes of nonneurogenic medication-refractory male BOO more precisely than PFS. The train or test division yielded similar proportions of ICS-BOO and VBOO classifications, supporting the reliability of the training set for model development and the test set for subsequent validation (Table 1).

### Performance Comparison Across 6 Machine Learning Algorithms

Both models 1 and 2 used 6 different algorithms for their predictive analyses, with comprehensive results presented in Table S1 in Multimedia Appendix 1. LR consistently achieved the highest AUC values for both models across the training and test sets, leading to its selection for developing both the nomogram and the web-based predictive tool.

### Model 1: ICS-BOO Prediction Nomogram Development and Performance

For model 1, a multivariate LR was used to construct a nomogram that predicts the probability of ICS-BOO. In developing this model, we included key clinical variables, with the final model retaining Qmax, Vvol, TPV, and PUA as contributory predictors. The odds ratios (ORs) for these variables were calculated, indicating that a decrease in Qmax and Vvol and an increase in TPV and PUA were associated with the presence of ICS-BOO. Specifically, the model detailed a statistically significant OR of 1.022 for each degree increase in PUA (*P*=.03) and 1.017 for each milliliter increase in TPV (*P*=.04), suggesting a strong influence on ICS-BOO risk (Table 2). The predictive capability of the model was further substantiated by a satisfactory Hosmer-Lemeshow test (*P*=.36) and by the calibration plot (Figure S1 in Multimedia Appendix 1), indicating a robust fit with the observed outcomes.

**Table 2:** ICS-BOO and VBOO prediction models using multivariate logistic regression.

Variables	Model 1^a^: ICS-BOO^b^ prediction			Model 2^c^: VBOO^d^ prediction	
	OR^e^ (95% CI)	*P* value	OR (95% CI)		*P* value
Age	—^f^	—	—		—
IPSS^g^-voiding	—	—	0.934 (0.871-0.999)		.05
IPSS-storage	—	—	—		—
IPSS-V/S^h^ ratio	—	—	—		—
Qmax^i^ (mL/s)	0.927 (0.858-1.002)	.06	0.908 (0.849-0.966)		.003
Voided volume (mL)	0.997 (0.993-1.001)	.10	1.004 (1-1.008)		.04
PVR^j^ (mL)	—	—	—		—
Total prostate volume (mL)	1.017 (1.001-1.034)	.04	—		—
Transitional zone index	—	—	—		—
IPP^k^ (cm)	—	—	2.253 (0.874-8.132)		.14
Prostatic urethral angle (º)	1.022 (1.002-1.042)	.03	1.017 (0.994-1.041)		.15

^a^Model-1: variable selection based on Akaike’s information criterion (223.45). Hosmer-Lemeshow goodness-of-fit test, *P*=.36.

^b^ICS-BOO: International Continence Society–defined bladder outlet obstruction.

^c^Model-2: variable selection based on Akaike’s information criterion (188.75). Hosmer-Lemeshow goodness-of-fit test, *P*=.54.

^d^VBOO: video-urodynamic studies–diagnosed bladder outlet obstruction.

^e^OR: odds ratio.

^f^The variable was not included in the model.

^g^IPSS: International Prostate Symptom Score.

^h^IPSS-V/S ratio: International Prostate Symptom Score voiding to storage score ratio.

^i^Qmax: maximum urinary flow rate.

^j^PVR: postvoid residual.

^k^IPP: intravesical prostatic protrusion.

**Figure 2:**
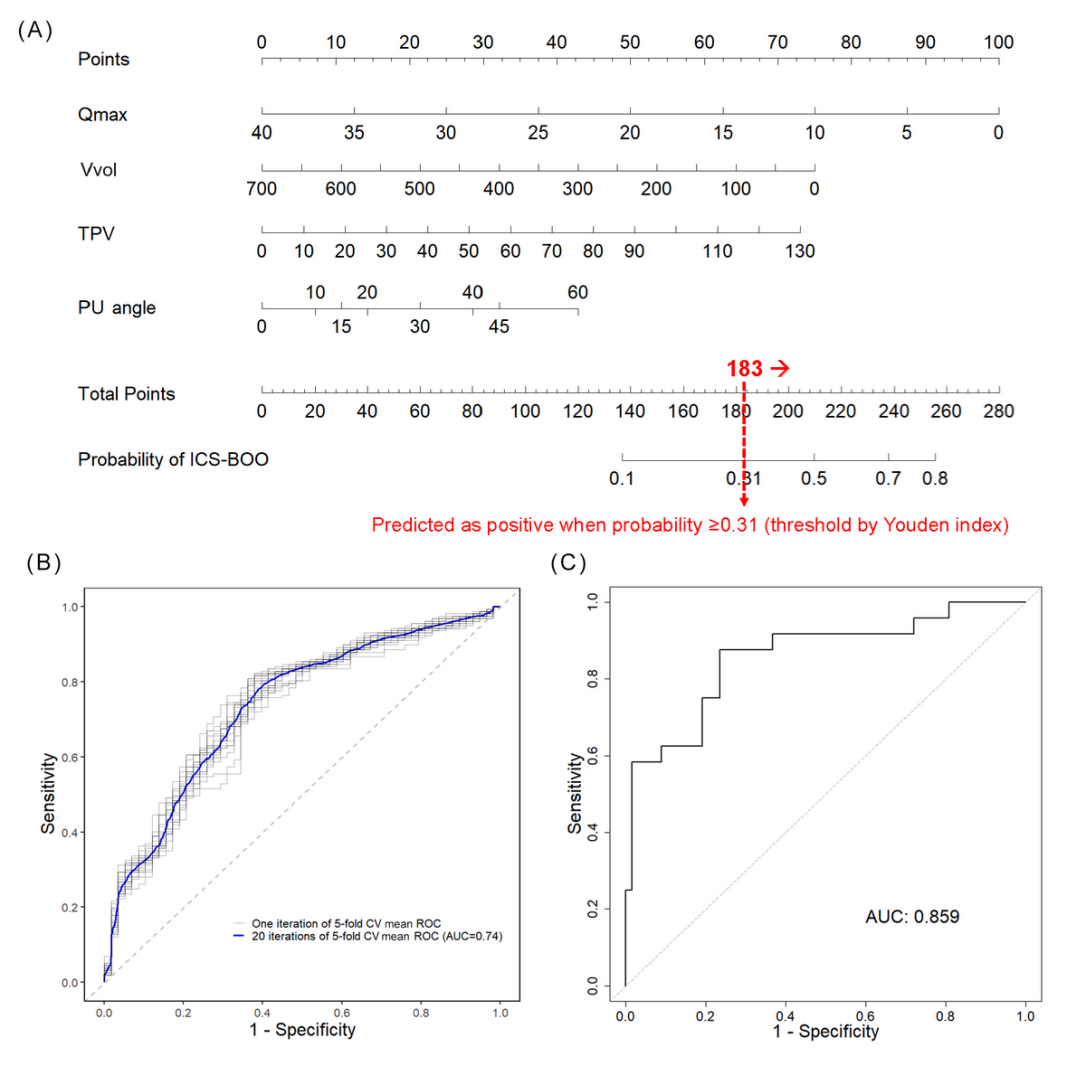
Nomogram for International Continence Society–defined bladder outlet obstruction prediction (model 1) with validation. (A) Nomogram for International Continence Society–defined bladder outlet obstruction prediction, (B) mean receiver operating characteristic curve of development validation by 20 iterations of 5-fold cross-validation on the training set, and (C) receiver operating characteristic curve of test validation on the test set. AUC: area under the receiver operating characteristic curve; CV: cross-validation; ICS-BOO: International Continence Society–defined bladder outlet obstruction; PU angle: prostatic urethral angle; Qmax: maximum urinary flow rate; ROC: receiver operating characteristic; TPV: total prostate volume; Vvol: voided volume.

The nomogram derived from model 1 was visually represented, plotting points for each variable that corresponded to their measured values. All variables, such as Qmax, Vvol, TPV, and PUA, were translated into points, which were then summed to yield a total score. This score was mapped to the corresponding probability of ICS-BOO, with the threshold for predicting a positive ICS-BOO outcome set at a probability of 0.31, as determined by the Youden index (Figure 2A).

Model 1, designed for ICS-BOO prediction, demonstrated solid performance across various metrics (Table 3 and Figure 2). During the development phase, the model achieved an AUC of 0.76 on the training data set, indicating a good ability to differentiate between the presence and absence of ICS-BOO. The development validation, which involved 20 repetitions of 5-fold cross-validation on the training set, reported an AUC of 0.74 (SD 0.09). The accuracy of the model was consistent as well, with a development validation accuracy of 0.76 (SD 0.04), slightly higher than the developmental phase accuracy of 0.75.

**Table 3:** Performance metrics of ICS-BOO and VBOO prediction models.

Performance metrics	Mode 1^a^: ICS-BOO^b^ prediction			Mode 2^c^: VBOO^d^ prediction		
	Development	Development validation	Test validation	Development	Development validation	Test validation
Data set cohort	Training	Training set 20x 5-fold CV^e^, mean (SD)	Test	Training	Training set 20x 5-fold CV, mean (SD)	Test
AUC^f^	0.76	0.74 (0.09)	0.86	0.75	0.71 (0.06)	0.72
Accuracy	0.75	0.76 (0.04)	0.77	0.80	0.77 (0.06)	0.76
Sensitivity	0.67	—^g^	0.75	0.85	—	0.87
Specificity	0.78	—	0.78	0.58	—	0.29
PPV^h^	0.53	—	0.55	0.90	—	0.84
NPV^i^	0.87	—	0.90	0.46	—	0.33
*F*_1_-score	0.59	—	0.63	0.87	—	0.85

^a^Binary classification best threshold: Youden index, *P*≥.31.

^b^ICSS-BOO: International Continence Society–defined bladder outlet obstruction.

^c^Binary classification best threshold: Youden index, *P*≥.73.

^d^VBOO: video-urodynamic studies–diagnosed bladder outlet obstruction.

^e^CV: cross-validation, presented as mean (SD).

^f^AUC: area under the receiver operating characteristic curve.

^g^Not available.

^h^PPV: positive predictive value.

^i^NPV: negative predictive value.

When applied to the external test set, model 1’s performance improved, with the AUC increasing to 0.86. The test validation also revealed a sensitivity of 0.75, a specificity of 0.78, a PPV of 0.55, and a notably high NPV of 0.90, along with an *F*_1_-score of 0.63, balancing PPV and sensitivity. To summarize, the robustness of the ICS-BOO prediction model was confirmed across both development and test validations.

### Model 2: Development and Performance of the VBOO Prediction Nomogram

For model 2, a multivariate LR was used to develop a nomogram that predicts the probability of VBOO. In the model selection process based on AIC, 5 variables, including IPSS-voiding (OR 0.934), Qmax (OR 0.908), Vvol (OR 1.004), IPP (OR 2.253), and PUA (OR 1.017) were retained in the final model as contributory predictors. Specifically, the model detailed statistically significant OR in Qmax (*P*=.003), Vvol (*P*=.04), and IPSS-voiding (*P*=.05), suggesting strong associations with VBOO risk (Table 2). The model demonstrated a satisfactory goodness of fit, as indicated by a Hosmer-Lemeshow test *P* value of .54 and by the calibration plot (Figure S2 in Multimedia Appendix 1).

**Figure 3:**
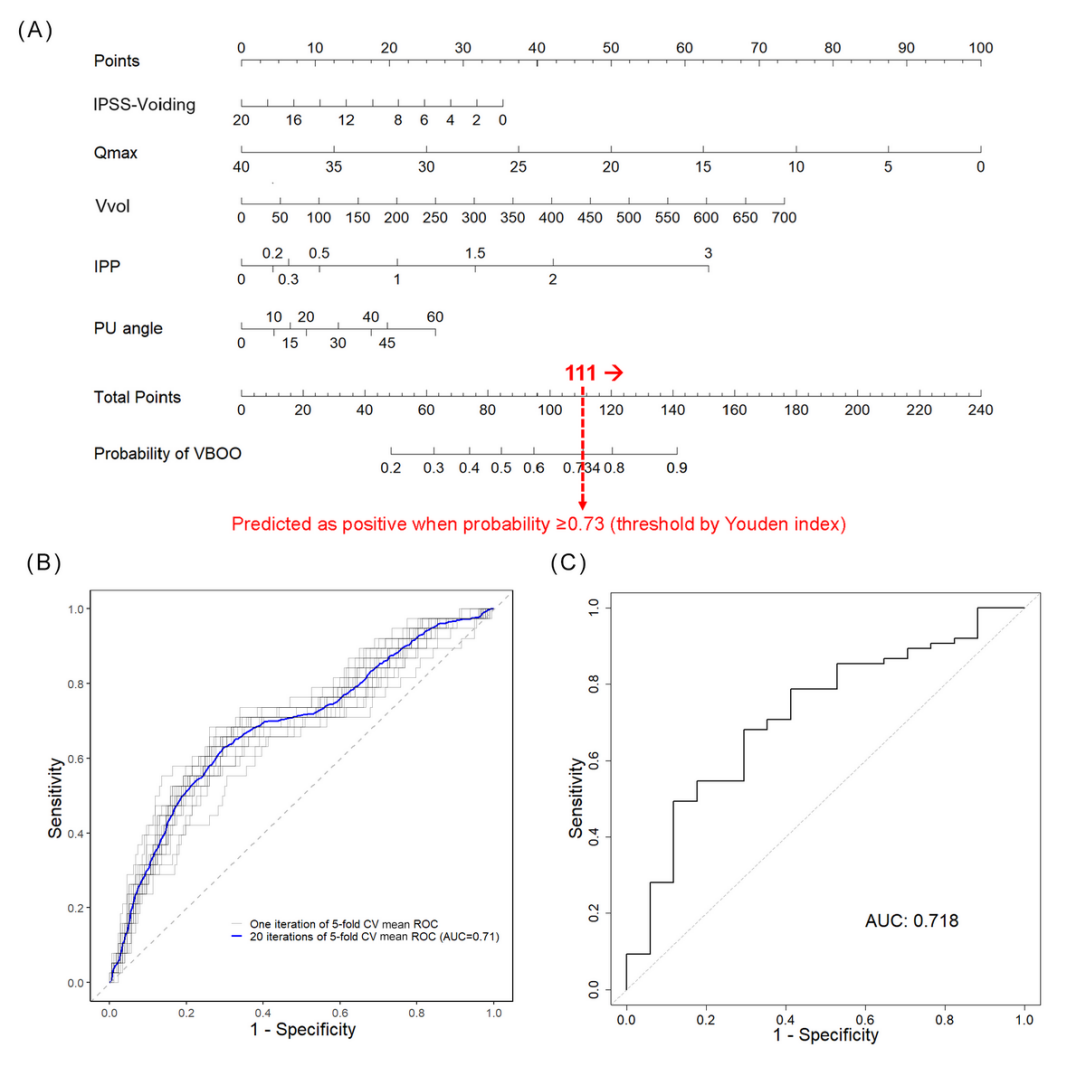
Nomogram for video-urodynamic studies–diagnosed bladder outlet obstruction prediction (model 2) with validation. (A) Nomogram for video-urodynamic studies–diagnosed bladder outlet obstruction prediction, (B) mean receiver operating characteristic curve of development validation by 20 iterations of 5-fold cross-validation on the training set, and (C) receiver operating characteristic curve of test validation on the test set. AUC: area under the receiver operating characteristic curve; CV: cross-validation; IPP: intravesical prostatic protrusion; IPSS: International Prostate Symptom Score; PU angle: prostatic urethral angle; Qmax: maximum urinary flow rate; ROC: receiver operating characteristic; VBOO: video-urodynamic studies–diagnosed bladder outlet obstruction; Vvol: voided volume.

The nomogram for model 2 translates these variables into a points system, allowing for an intuitive prediction of VBOO risk. For instance, a decrease in IPSS-voiding or Qmax scores increases the risk points on the nomogram, directly correlating with a higher probability of VBOO. The nomogram defines a positive prediction for VBOO when the probability is ≥0.73, as determined by the Youden index. This threshold was chosen to ensure the nomogram effectively balances sensitivity and specificity (Figure 3A).

Model 2, aimed at predicting VBOO, exhibited robust performance in both the development and validation phases (Table 3 and Figure 3). During the initial development of the training data set, the model achieved an AUC of 0.75, indicating respectable discriminative ability. The accuracy was notably high at 0.80, with an outstanding sensitivity of 0.85, suggesting the model’s effectiveness in identifying patients with VBOO. The specificity, however, was moderate at 0.58, which is reflected in the high PPV of 0.90 and a lower NPV of 0.46. The *F*_1_-score, which harmonizes the PPV and sensitivity, stood at 0.87, highlighting the model’s balanced performance.

In the development validation, model 2 maintained an AUC of 0.71 (SD 0.06) and an accuracy of mean 0.77 (SD 0.06), confirming the model’s consistency. The test validation on the test set further validated the model, with an AUC of 0.72. The sensitivity was notably high at 0.87 on the test set, surpassing the training performance. Despite this, the external PPV remained high at 0.84, and the NPV improved to 0.33, with an *F*_1_-score comparable to the development phase at 0.85.

### Dual Model Predictions Aiding Clinical Decision-Making

**Figure 4:**
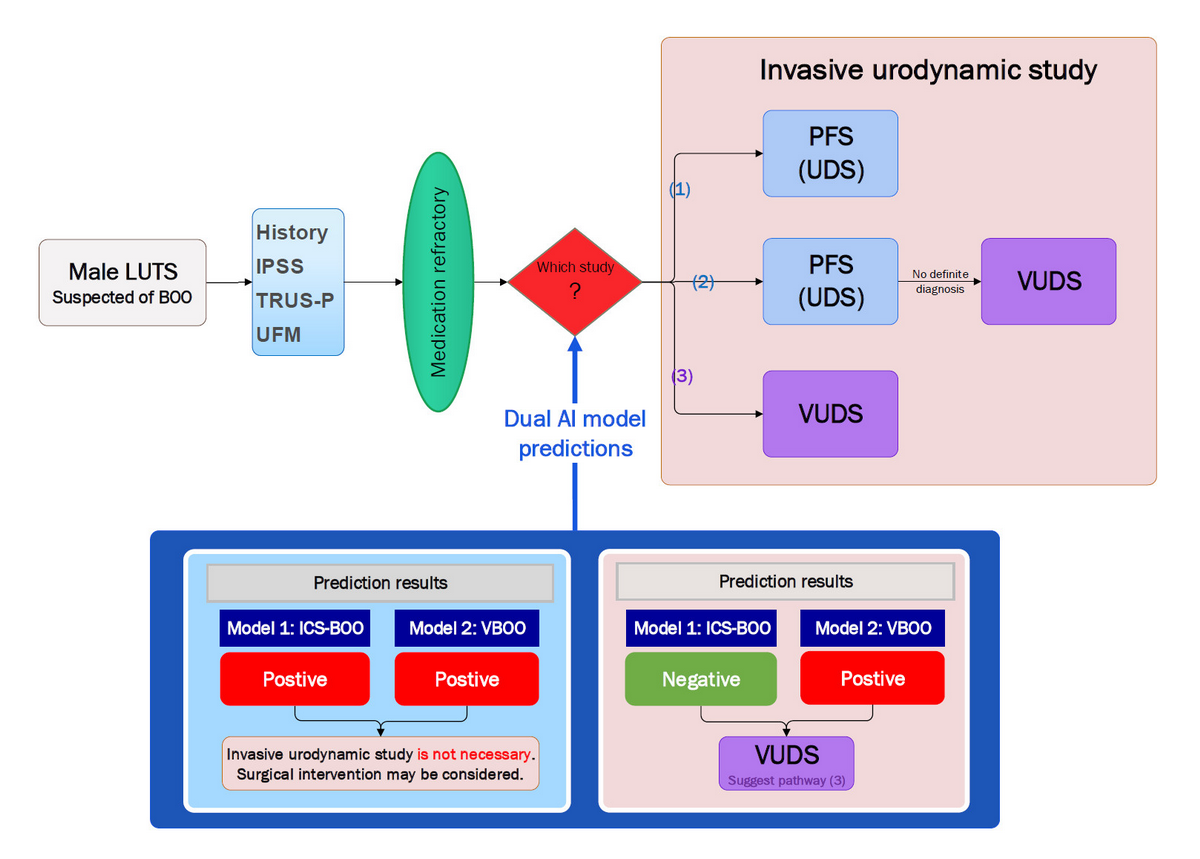
Algorithmic flowchart of dual artificial intelligence models predicting bladder outlet obstruction to aid clinical decision-making for nonneurogenic male lower urinary tract symptoms. From the clinical decision-making perspective, there exists a dilemma for men with medication-refractory nonneurogenic lower urinary tract symptoms who are suspected of having bladder outlet obstruction. The following question arises: which diagnostic urodynamic study is most appropriate for the patient? (1) Should one take the first pathway and perform pressure-flow studies only to potentially obtain an accurate diagnosis? (2) Or should one follow the second pathway, starting with pressure-flow studies and proceeding to video-urodynamic studies if a definitive diagnosis is not achieved? (3) Alternatively, is the third pathway preferable, where one directly conducts video-urodynamic studies for a detailed diagnosis? By the dual-model prediction approach, if both models positively predict bladder outlet obstruction, suggesting it is caused by either medication-refractory primary bladder neck obstruction or benign prostatic obstruction, surgical intervention may be considered; thus, video-urodynamic studies or pressure-flow studies might be unnecessary. Conversely, when prediction for International Continence Society–defined bladder outlet obstruction is negative but video-urodynamic studies–diagnosed bladder outlet obstruction is positive, indicating varied etiology, video-urodynamic studies are advised over pressure-flow studies for precise diagnosis and guiding subsequent therapy. AI: artificial intelligence; BOO: bladder outlet obstruction; ICS-BOO: International Continence Society–defined bladder outlet obstruction; IPSS: International Prostate Symptom Score; LUTS: lower urinary tract symptoms; PFS: pressure-flow studies; TRUS-P: transrectal ultrasound of the prostate; UDS: urodynamic study; UFM: uroflowmetry; VBOO: video-urodynamic studies–diagnosed bladder outlet obstruction; VUDS: video-urodynamic studies.

The preceding results demonstrate that VUDS can more accurately differentiate between causes of nonneurogenic male BOO compared with PFS. Although an initial attempt was made to develop a single AI model using noninvasive parameters to predict the exact VUDS diagnosis (a 5-class classification), it resulted in poor accuracy (Multimedia Appendix 1). However, 2 ML models designed to predict ICS-BOO and VBOO in patients with medication-refractory, nonneurogenic male LUTS have shown commendable discrimination performance. When the predictions from both models are applied to the same patient, their combined insights can significantly enhance clinical decision-making and simplify the diagnostic pathway. The algorithm detailing this combined predictive approach is depicted in Figure 4.

In the validation performed on the test set (n=92), diagnoses were concurrently made for each patient using the ICS-BOO and VBOO criteria, resulting in an actual confusion matrix as illustrated in Figure S3 in Multimedia Appendix 1. In addition, using the dual model prediction approach produced a separate dual predictions confusion matrix. The comparison revealed a similar distribution of BOO across the 2 confusion matrices, suggesting a substantial agreement between the actual BOO diagnoses and the predictions from both the ICS-BOO and VBOO models.

In our analysis, both the ICS-BOO and VBOO models predicted positively in 30 out of 92 patients, which represents 32.6% of the test cohort. Notably, for these 30 patients, the BOO was determined to be exclusively caused by either BPO or PBNO, accounting for 100% of cases within this subgroup, with no cases diagnosed as DV. For this subgroup of patients with medication-refractory BPO or PBNO, similar surgical interventions such as transurethral resection of the prostate (TURP) or transurethral incision of the prostate (TUIP) may be suggested, thus guiding the subsequent surgical treatment. Consequently, VUDS could potentially be avoided for these patients, aiding in the diagnosis of male BOO and possibly reducing the necessity of such invasive examinations for approximately one-third of the test cohort.

Conversely, in the test cohort of 92 patients, there were 51.1% (n=47) cases where the ICS-BOO model predicted negatively while the VBOO model predicted positively. Within this particular subgroup, the actual diagnoses were divergent—PBNO was identified in 40% (19/92) cases, BPO in 19% (9/92) cases, DV in 15% (7/92) cases, and non-BOO in 26% (12/92) cases. Therefore, for this subgroup, the use of VUDS rather than PFS is strongly recommended for differential diagnosis, which guides varied treatment, thereby precisely targeting 51.1% (47/92) of patients for VUDS (Figure 4).

### Web-Based Dual-Model Prediction Tool

**Figure 5:**
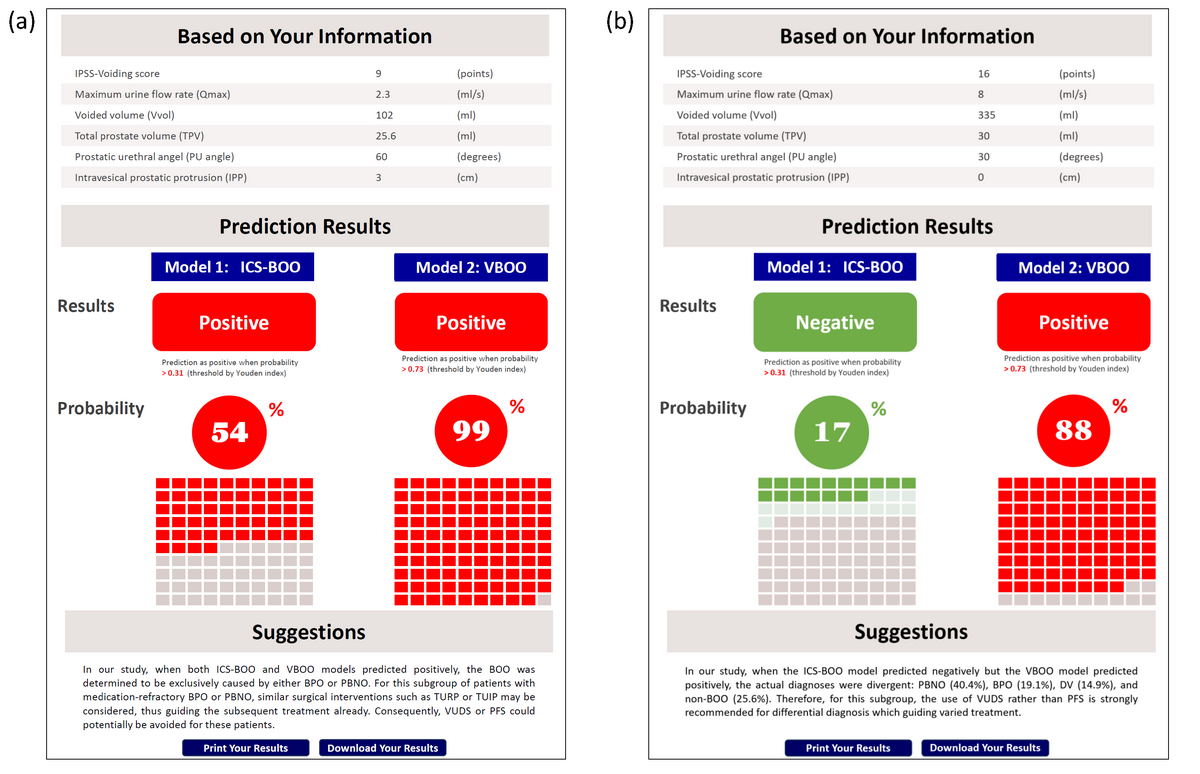
Two screenshots of outputs from the web-based dual-model prediction tool. Based on 6 noninvasive clinical parameters from 2 different patients, the prediction results are as follows: (A) demonstrates a scenario where both International Continence Society–defined bladder outlet obstruction and video-urodynamic studies–diagnosed bladder outlet obstruction models predict a positive outcome for bladder outlet obstruction, with probabilities of 54% and 99%, respectively, indicating a high likelihood of bladder outlet obstruction caused by either benign prostatic obstruction or primary bladder neck obstruction and suggesting that invasive studies like video-urodynamic studies or pressure-flow studies may be unnecessary. (B) illustrates a case where the International Continence Society–defined bladder outlet obstruction model predicts a negative outcome (17% probability) while the video-urodynamic studies–diagnosed bladder outlet obstruction model predicts a positive outcome (88% probability), suggesting a complex bladder outlet obstruction etiology that may not present with high-pressure low-flow dynamics and may benefit from further evaluation with video-urodynamic studies. BOO: bladder outlet obstruction; BPO: benign prostatic obstruction; DV: dysfunctional voiding; ICS-BOO: International Continence Society–defined bladder outlet obstruction; IPSS: International Prostate Symptom Score; PBNO: primary bladder neck obstruction; PFS: pressure-flow studies; TUIP: transurethral incision of the prostate; TURP: transurethral resection of the prostate; VBOO: video-urodynamic studies–diagnosed bladder outlet obstruction; VUDS: video-urodynamic studies.

We developed a user-friendly web-based online prediction tool that simplifies the dual-model prediction process for BOO. This tool unifies the predictors of both models into 6 parameters, listed as IPSS-voiding score, Qmax, Vvol, TPV, PUA, and IPP, enabling clinicians to input patient-specific data and instantly receive probabilities for both ICS-BOO and VBOO with corresponding decision-making recommendations into one output. This innovation seamlessly incorporates AI-driven predictions into the clinical workflow, helping to discern which patients may require a surgical intervention or a VUDS, thereby facilitating and optimizing the precision of patient care (Figure 5).

Figure 5 demonstrates 2 screenshots of outputs from the web-based dual-model prediction tool. Using 6 noninvasive clinical parameters from 2 different patients, the tool generates the following predictive results: in Figure 5A, both ICS-BOO and VBOO models make a positive BOO prediction, with probabilities of 54% and 99%, respectively, indicating a high likelihood of BOO caused by either BPO or PBNO and suggesting that surgical intervention may be considered and invasive studies like VUDS or PFS may be unnecessary. Figure 5B illustrates a case where the ICS-BOO model predicts a negative outcome (17% probability) while the VBOO model predicts a positive outcome (88% probability), suggesting a complex BOO etiology that may not present with classical high-pressure low-flow dynamics and may benefit from further evaluation with VUDS (Multimedia Appendix 1).

## Discussion

### Uniqueness and Contributions

The uniqueness of this study lies in using VUDS to identify the causes of male BOO and LUTS. In the literature review, most studies only use PFS as the standard method for assessing BOO. Our model 1 also adopts this conventional approach by using a BOOI greater than 40 as the diagnostic standard. In contrast, this study uses the more accurate VUDS as the diagnostic standard for BOO in our model 2, enabling the identification of more patients with potential obstructions. Using these data, 2 predictive models are established. To our knowledge, no other studies have been conducted in this area.

Male BOO is a urodynamic condition in which a bladder outlet cannot open during urination. The underlying pathophysiology could be anatomical (due to BPO or US) or functional (PBNO or DV) obstruction. The results revealed that VUDS excels in identifying the causes of nonneurogenic medication-refractory male BOO more precisely than PFS, as evidenced by the higher prevalence of BOO diagnoses made by VUDS (252/307, 82.1%) compared to those identified by PFS (82/307, 26.7%; *P*<.001). For example, patients exhibiting a nonrelaxing bladder neck during emptying—a type of BOO that could only be diagnosed through VUDS, specifically type 2 PBNO [22]—when combined with low Pdet.Qmax might have a BOOI under 40. This falls short of the ICS-BOO standards, according to PFS, leading to misclassification as non-BOO. Such complicated etiologies of male BOO can only be determined by VUDS but not by PFS alone.

Alternatively, our innovative VBOO model, used before any invasive UDS, can identify potential BOO when predictions are positive. Alongside ICS-BOO predictions, whether positive or negative, it aids in determining the need for surgical intervention or further VUDS evaluation. When both VBOO and ICS-BOO predictions are positive, indicating medication-refractory PBNO or BPO, similar surgical interventions such as TURP or TUIP may be considered, thus guiding the subsequent treatment. Therefore, an invasive VUDS or PFS examination might be unnecessary.

Conversely, a positive VBOO prediction with a negative ICS-BOO suggests a complex BOO etiology not typically presenting with high-pressure, low-flow dynamics, possibly indicating a BOOI <40 or within the BOOI equivocal zone, necessitating further VUDS evaluation for these patients. Furthermore, given the high sensitivity (0.86) of the VBOO model, a negative VBOO prediction from such a sensitive test can reliably exclude the BOO disease (as non-BOO), a principle commonly referred to as SnNOUT [23].

The ICS-BOO standard, defined by a BOOI >40, represents the classic high-pressure low-flow pattern but may not capture all obstructive disorders. For example, BOOI <20 indicates nonobstruction, while values between 20 and 40 fall into an “equivocal” zone by the ICS BOOI standard, where PFS alone may not definitively classify a patient’s condition. This is depicted in the clinical decision-making “pathway (2)” in Figure 4, where the patients with medication-refractory male LUTS who do not receive a definitive diagnosis from PFS may require further VUDS examination—a situation that occurs in real-world practice and can lead to multiple invasive examinations for the patient. Hence, our VBOO prediction model serves as a clinical decision support tool, particularly beneficial for physicians or hospitals lacking VUDS facilities, aiding treatment decisions based on noninvasive predictions.

Our dual nomograms were developed for and applied to a distinct patient cohort: nonneurogenic patients with medication-refractory male LUTS suspected of BOO, differentiating them from men with treatment-naive or typical LUTS. This specific focus aligns our application more closely with real clinical decision-making scenarios. Typically, PFS or VUDS are not routinely performed for all men with common LUTS but are considered when initial diagnoses and medication trials are ineffective. This approach suggests that the pathophysiology in our patient group might be more complex than in common LUTS cases. A considerable portion might have had other functional BOO pathologies such as PBNO or DV, or non-BOO causes such as DO, DU, or BO. It was evidenced by the fact that our cohort showed a relatively low incidence of ICS-BOO (82/307, 26.7%) compared to the prevalence in common patients with male LUTS, yet a high prevalence of VBOO (252/307, 82.1%). Therefore, this study, which used a relatively challenging-to-diagnose cohort to validate the performance of our ICS-BOO model, should not be directly compared in terms of performance with other nomograms validated using patients with common LUTS [16,24].

### Noninvasive Predictors

The ICS-BOO model incorporates Qmax, Vvol, PUA, and TPV as predictors, while the VBOO model includes Qmax, Vvol, PUA, IPSS-voiding, and IPP. Thus, these 2 models share 3 common predictors: Qmax, Vvol, and PUA. Qmax was significantly associated with the presence of ICS-BOO and VBOO, aligning with earlier research on male treatment-naïve or common LUTS [11]. However, the diagnostic reliability of Qmax alone is limited, as conditions like DU may also result in a lower Qmax, thus complicating the prediction of BOO based solely on this parameter [25]. Similarly, the PUA was incorporated into both ICS-BOO and VBOO models showing a positive correlation between the angle degree and the likelihood of BOO, consistent with findings from previous studies [26-28]. Ku et al [28] revealed that patients with higher PUA had a higher BOOI than those with lower PUA, suggesting PUA’s utility in PFS-obstruction prediction. Moreover, PUA also contributes to our VBOO prediction, thus indicating that PUA may play an important role associated with the varied etiologies of BOO. Regarding TPV, which is significantly associated with the ICS-BOO model but not included as a predictor in the VBOO model, it suggests that a high TPV is correlated with a high BOOI, aiding in inferring that the etiology could be BPO [29]. Conversely, TPV’s lack of contribution to VBOO prediction reasonably implies that it is not useful in detecting functional BOO such as PBNO or DV. Finally, the inclusion of the IPSS-voiding score, rather than the IPSS-storage score, in the VBOO model’s prediction aligns well with domain knowledge [30,31].

### Clinical Significance

In summary, our dual-model AI application is a pioneering tool in functional urology. The strengths and medical significance of the application are outlined as follows:

1. Aiding clinical decision-making and guiding treatment: our dual-model approach uses noninvasive clinical parameters to predict anatomical or functional BOO, providing actionable recommendations and reducing the need for invasive procedures like VUDS or PFS. Specifically:

- When both ICS-BOO and VBOO predictions are positive, it suggests that surgical interventions such as TURP or TUIP may be considered without further invasive studies.
- A negative ICS-BOO but positive VBOO prediction suggests using VUDS over PFS for differential diagnosis, guiding varied treatment approaches.
- A negative prediction in both models reliably excludes BOO, prompting the evaluation of other non-BOO conditions such as DU, DO, or BO, guiding appropriate treatment.

2. Reduction of invasive procedures: the application potentially reduces the frequency of unnecessary invasive tests by about one-third, enhancing patient comfort and reducing complications and health care costs.

3. Streamlining clinical workflows: the web-based AI tool integrates seamlessly into clinical workflows, enabling quick, data-driven decision-making and personalized patient management.

4. Expanding access to advanced diagnostics: in environments lacking VUDS due to cost or logistical constraints, our tool offers a practical alternative, democratizing advanced diagnostics and potentially improving outcomes.

5. Optimizing treatment for challenging cases: our application aids in managing challenging clinical scenarios, such as those involving patients with medication-refractory male LUTS who have experienced primary treatment failure. A lack of accurate diagnosis in such complex cases can lead to inappropriate treatment and poor outcomes. For example, performing TURP surgery on a patient suspected of BOO could fail to improve symptoms if the actual diagnosis is DV rather than BPO. Our application helps identify which patients might benefit directly from surgery without the need for invasive VUDS or PFS, thus optimizing treatment strategies and improving patient outcomes.

6. Innovative approach over existing tools: unlike traditional models that depend solely on PFS, this study uses VUDS data, addressing the limitations of existing models in handling equivocal BOOI zones. Our dual-model prediction approach represents an innovative advancement in this field. The combination of ICS-BOO and VBOO models sufficiently aids clinical decision-making and guides treatment, surpassing the capabilities of traditional single-prediction models based solely on ICS-BOO criteria.

7. Transparent and interpretable AI models: after fine-tuning the hyperparameters of 6 ML algorithms, LR consistently achieved the highest AUC values for both models. The LR model’s selected predictors and their corresponding ORs offer high clinical interpretability, which enhances trust and usability among clinicians.

8. Flexible use from single to dual models, traditional to modern approaches: our tool offers flexibility in clinical application, enabling clinicians to adopt only a single model or a dual-model approach based on their preference. In addition, the paper-based nomogram provides a traditional, user-friendly option that is adaptable for various clinical scenarios.

### Limitations

This study has several limitations. First, its retrospective design introduces a potential for selection bias due to the exclusion of patients with incomplete data. Second, using data solely from a single medical institution might limit the generalizability of the findings. To enhance the robustness of our predictive model, future validation with multicenter data is essential. Furthermore, the models developed specifically for patients with medication-refractory, nonneurogenic LUTS might not be suitable for those with initial diagnoses or common LUTS. In addition, although a range of parameters were considered as predictive factors, not including concurrent comorbidities may have limited the control over potential confounding factors.

### Conclusions

Nonneurogenic male BOO caused by BPO, PBNO, DV, or US cannot be precisely differentiated solely through PFS or a single AI prediction model. However, 2 ML models predicting ICS-BOO and VBOO among patients with medication-refractory, nonneurogenic male LUTS using noninvasive clinical parameters demonstrate commendable discrimination performance. By our innovative dual-model prediction approach, if both models positively predict BOO, suggesting it is caused by either medication-refractory PBNO or BPO, surgical intervention may be considered, thus VUDS might be unnecessary for about one-third of patients. Conversely, when prediction for ICS-BOO is negative but VBOO is positive, indicating varied etiology, VUDS is advised over PFS for precise diagnosis and guiding subsequent therapy, accurately identifying 51.1% (47/92) of patients for VUDS.

## Appendix

Multimedia Appendix 1 Additional material.

Multimedia Appendix 2 Data S1: Dataset for validation of ICS-BOO and VBOO models.

## Abbreviations

AI: artificial intelligence
AIC: Akaike’s information criterion
AUC: area under the receiver operating characteristic curve
BOO: bladder outlet obstruction
BOOI: bladder outlet obstruction index
BPO: benign prostatic obstruction
DO: detrusor overactivity
DU: detrusor underactivity
DV: dysfunctional voiding
ICS: International Continence Society
ICS-BOO: International Continence Society–defined bladder outlet obstruction
IPP: intravesical prostatic protrusion
IPSS: International Prostate Symptom Score
LR: logistic regression
LUTS: lower urinary tract symptoms
ML: machine learning
NPV: negative predictive value
OR: odds ratio
PBNO: primary bladder neck obstruction
Pdet.Qmax: maximum detrusor pressure at Qmax
PFS: pressure-flow studies
PPV: positive predictive value
PUA: prostatic urethral angle
Qmax: maximum urinary flow rate
TPV: total prostate volume
TUIP: transurethral incision of the prostate
TURP: transurethral resection of the prostate
UDS: urodynamic study
US: urethral stricture
VBOO: video-urodynamic studies–diagnosed bladder outlet obstruction
VUDS: video-urodynamic studies
Vvol: voided volume

## Data Availability

The data sets generated and/or analyzed during the current study are available from the corresponding author upon reasonable request.
