# Supplementary material for: Building Dual AI Models and Nomograms Using Noninvasive Parameters for Aiding Male Bladder Outlet Obstruction Diagnosis and Minimizing the Need for Invasive Video-Urodynamic Studies: Development and Validation Study
Source: J Med Internet Res. 2024 Jul 23;26:e58599. doi: 10.2196/58599 (PMC11303901; doi:10.2196/58599)
Supplement: Multimedia Appendix 1 [file jmir_v26i1e58599_app1.pdf]

SUPPLEMENTARY MATERIALS

Contents

I. **Table S1**: Performance of Model 1 and 2 Using Six Machine Learning Algorithms

II. **Figure S1:** Calibration plot of ICS-BOO Prediction

III. **Figure S2**: Calibration plot of VBOO Prediction

IV. **Figure S3:** Dual Model Predictions Confusion Matrix

V. **Document S1:** Medication-Refractory Male LUTS

VI. **Document S2:** Single Multiclass Prediction Model for VUDS diagnosis

VII. **Document S3:** Prototype and Test Version of the Web-based Prediction Tool

VIII. **Data S1:** Dataset for Validation of ICS-BOO and VBOO Models.xlsx

I. Table S1. Performance of Model 1 and 2 Using Six Machine Learning Algorithms

Table S1. Predictive Performance of Model 1 and 2 on the Test Dataset Using Six Machine Learning Algorithms

Algorithms	AUC	Accuracy	Sensitivity	Specificity	PPV	NPV	F1-score	Optimal Threshold
**Model 1: ICS-BOO**								
LR	**0.86**	0.77	0.75	0.78	0.55	0.9	0.63	0.31
SVM	0.86	0.82	0.67	0.87	0.64	0.88	0.65	0.30
DT	0.73	0.71	0.75	0.69	0.46	0.89	0.57	0.28
RF	0.83	0.76	0.71	0.78	0.53	0.88	0.61	0.29
GB	0.75	0.75	0.75	0.75	0.51	0.89	0.61	0.295
XGB	0.82	0.79	0.75	0.81	0.58	0.9	0.65	0.38
**Model 2: VBOO**								
LR	**0.72**	0.76	0.87	0.29	0.84	0.33	0.85	0.73
SVM	0.71	0.66	0.68	0.59	0.88	0.29	0.77	0.81
DT	0.68	0.57	0.49	0.88	0.95	0.28	0.65	0.93
RF	0.58	0.77	0.92	0.12	0.82	0.25	0.87	0.61
GB	0.58	0.71	0.85	0.06	0.8	0.08	0.83	0.99
XGB	0.53	0.73	0.85	0.18	0.82	0.21	0.84	0.65

The metrics assessed are area under the receiver operating characteristic curve (AUC), positive predictive value (PPV) and negative predictive value (NPV). The optimal threshold was determined by the Youden Index. The algorithms compared include logistic regression (LR), support vector machine (SVM), decision tree (DT), random forest (RF), gradient boosting (GB), and extreme gradient boosting (XGB).

- AUC represents the probability that a randomly chosen positive instance is ranked higher than a negative one, reflecting the overall performance of the model.
- Sensitivity is the true positive rate, highlighting the model's detection capability.
- Specificity is the true negative rate, showing the model's ability to identify negatives.
- PPV is the proportion of positive test outcomes that are true positives, assessing precision.
- NPV is the proportion of negative test outcomes that are true negatives, showing true negative identification efficiency.

II. Figure S1: Calibration plot of ICS-BOO Prediction

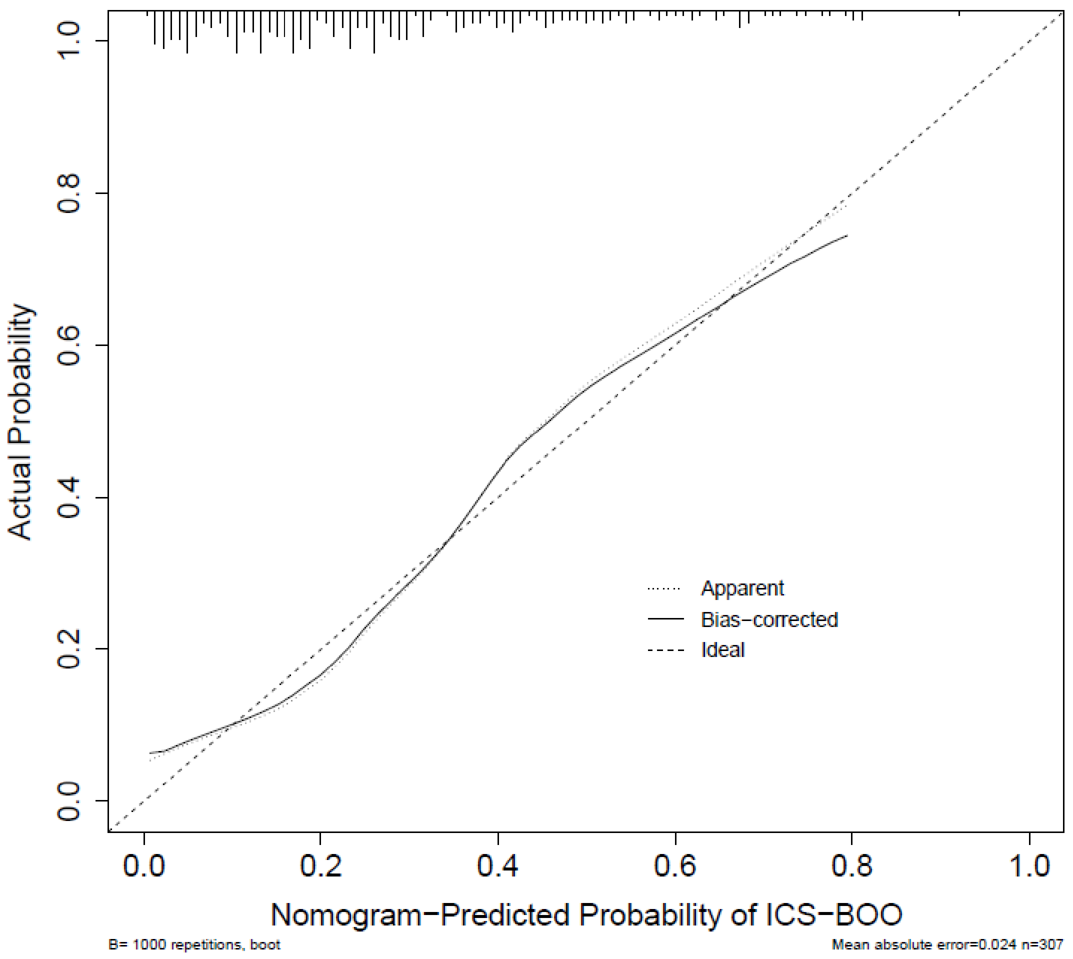

Figure S1: Calibration plot of ICS-BOO Prediction (Model1)

Calibration plots, which visually compare predicted probabilities with observed outcomes, thus ensuring that the model's predictions align closely with actual risk.

ICS-BOO: International Continence Society-defined BOO

III. Figure S2: Calibration plot of VBOO Prediction

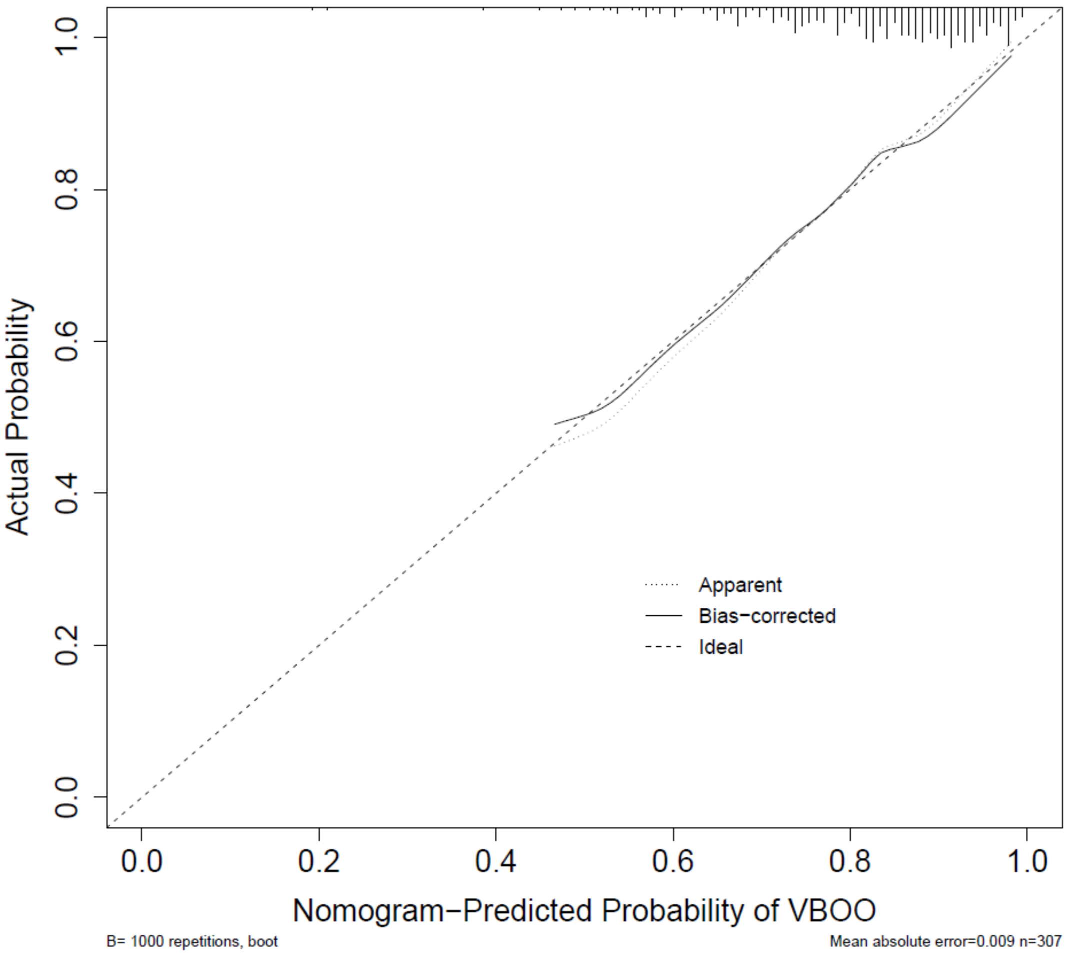

Figure S2: Calibration plot of VBOO Prediction (Model2)

Calibration plots, which visually compare predicted probabilities with observed outcomes, thus ensuring that the model's predictions align closely with actual risk.

VBOO: VUDS-diagnosed BOO

IV. Figure S3. Dual Model Predictions Confusion Matrix

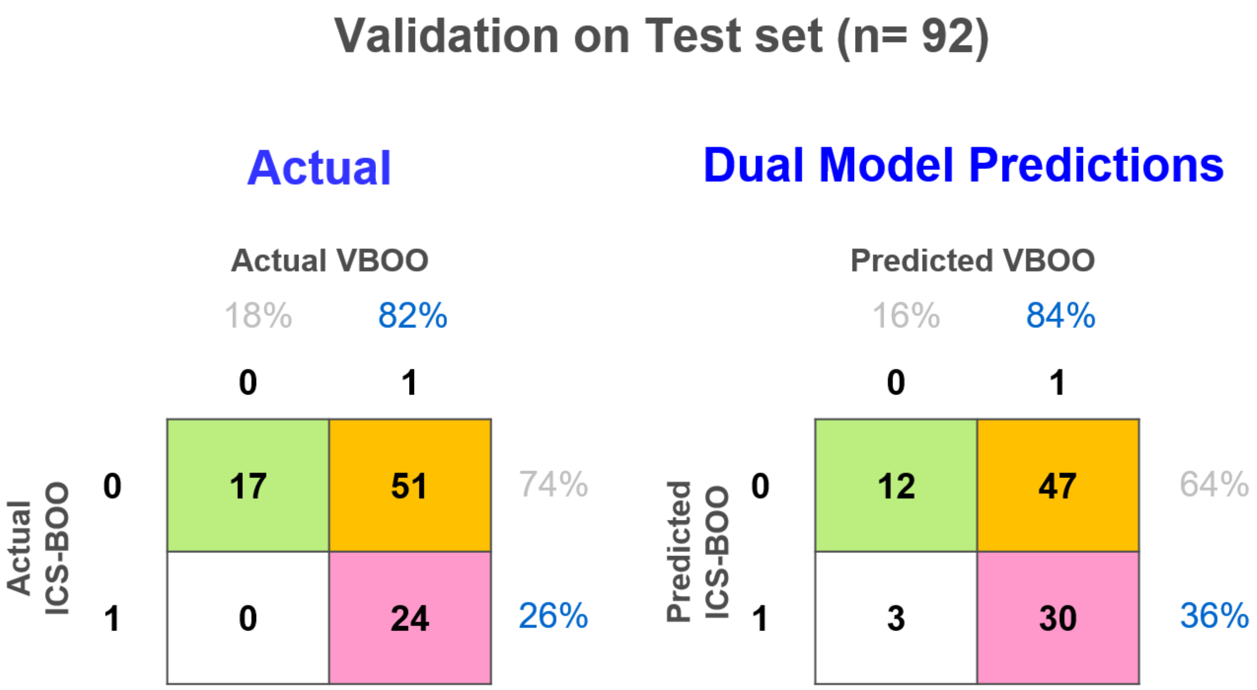

'0' absence of disease (negative); '1' presence of disease (positive)

Figure S3. ICS-BOO vs VBOO Confusion Matrix based on Actual diagnosis and Dual model predictions

Diagnoses were concurrently made for each patient using the ICS-BOO and VBOO criteria, resulting in an Actual confusion matrix. Furthermore, employing the dual model predictions approach on the test set produced a Dual Predictions confusion matrix.

This suggests that the dual model approach is fairly consistent with the actual diagnoses made using the ICS-BOO and VBOO criteria. Overall, the predictive models have demonstrated substantial agreement with the actual diagnoses, which could potentially streamline the diagnostic process for BOO.

V. Document S1: Medication-Refractory Male LUTS

Medication-refractory male LUTS is defined as a lack of symptomatic response to medical treatment over a continuous three-month period, a duration that aligns with clinical practice. Given that alpha-blockers and antimuscarinics typically exhibit a rapid onset, with symptom relief often within a week if effective, a three-month timeline ensures that patients have had multiple outpatient clinic evaluations, allowing for adequate trials of various monotherapies or combination therapies.

All medications referenced are consistent with those outlined in Section 5.2 of the EAU guideline on pharmacological treatment. The specific medications and their ATC codes are detailed in the accompanying table, providing clarity on the pharmacological interventions considered. α1-blockers are usually considered the first-line drug treatment for male LUTS because of their rapid onset of action, good efficacy, and low rate and severity of adverse events [1].

[1] Management of Non-neurogenic Male LUTS, EAU guideline. https://uroweb.org/guidelines/management-of-non-neurogenic-male-luts/chapter/disease-management

Male LUTS Medication	ATC code
**α-blockers**	
Tamsulosin	G04CA02
Doxazosin	C02CA04
Silodosin	G04CA04
Terazosin	G04CA03
**5α-reductase inhibitors (5ARI)**	
Finasteride	G04CB01
Dutasteride	G04CB02
Dutasteride&Tamsulosin	G04CA52
**Anti-muscarinics**	
Tolterodine	G04BD07
Solifenacin	G04BD08
Oxybutynin	G04BD04

VI. Document S2: Single Multiclass Prediction Model for VUDS diagnosis

Although an initial attempt was made to develop a single AI model utilizing non-invasive parameters to predict the exact VUDS diagnosis (a 5-class classification), it resulted in poor accuracy Multiclass classification involves predicting multiple categories, representing the correct class for each sample. In this study, five categories of bladder outlet obstruction - BPO, PBNO, PRES, DV, and non-BOO - were evaluated for predictive performance. Due to the lower sample sizes for PRES and DV, the dataset exhibited class imbalance. To address this, we utilized the Logistic Regression function from sklearn to adjust class weights for the imbalanced dataset. Different parameter settings were experimented with, including setting the class weight to 'balanced' and 'None', choosing between 'ovr' (one-vs-rest) and 'multinomial' for the classification method, as well as the default setting 'auto'.

After adjusting various parameter combinations, the following settings were selected: (1) 'class_weight' set to 'balanced' to automatically equalize class weights, (2) the classification method set to 'ovr', employing a one-vs-rest strategy, and the performance metrics calculated using micro-averaging.

From the multiclass classification metrics for BOO, it is observed that most specificities and negative predictive values (NPVs) reach as high as 0.8, seemingly capable of accurately distinguishing and predicting patients without BOO. However, most sensitivities are below 0.5, indicating that the model fails to successfully detect the majority of patients with BOO. Additionally, the positive predictive values (PPVs) do not exceed 0.6, suggesting that less than 60% of patients predicted to have BOO actually do, indicating a relatively poor outcome. Another crucial metric, the highest F1 score achieved is only 0.53, and the overall micro-average accuracy of the model stands at 0.34. Clearly, the overall performance of the multiclass BOO prediction model is suboptimal, as detailed in Table S2-2

Table S2-1. Confusion Matrix of 5-class VUDS Diagnosis Prediction

		Predicted				
		non-BOO	PBNO	BPO	PRES	DV
Actual	non-BOO	2	4	2	0	9
	PBNO	3	12	7	4	10
	BPO	3	3	13	2	5
	PRES	1	2	0	1	2
	DV	2	1	1	0	3

Table S2-2. Performance Metrics of 5-class VUDS Diagnosis Prediction

Classes	Sensitivity	Specificity	PPV	NPV	F1-score	Accuracy
non-BOO	0.12	0.88	0.18	0.81	0.14	
PBNO	0.33	0.82	0.55	0.66	0.41	
BPO	0.50	0.85	0.57	0.81	0.53	0.34
PRES	0.17	0.93	0.14	0.94	0.15	
DV	0.43	0.69	0.10	0.94	0.17	

VII. Document S3: Prototype and Test Version of the Web-based Prediction Tool

Regarding the availability of a public tool for validation, we have provided a dataset in the supplementary materials (**Data S1**) and a test version of the web-based prediction tool, allowing external parties to validate our nomograms and the Web-based tool. Although this prototype web tool is fully functional in terms of model prediction capabilities, its user interface is simplified compared to the final version, which features optimized graphical visualizations to enhance user experience, as illustrated in Figure 5. The final version of the tool has been developed and is hosted by a medical institution. Due to hospital regulations, the official site will not be publicly accessible until after the publication of this paper.

1. The prototype and test version of the web-based prediction tool, http://13.55.12.42/
2. You can download the supplementary dataset (Data S1):
   [Dataset for Validation of ICS-BOO and VBOO Models.xlsx]
